# Modelling soil organic carbon at multiple depths in woody encroached grasslands using integrated remotely sensed data

**DOI:** 10.1007/s10661-025-13671-w

**Published:** 2025-03-01

**Authors:** Sfundo Mthiyane, Onisimo Mutanga, Trylee Nyasha Matongera, John Odindi

**Affiliations:** 1 Discipline of Geography and Environmental Science, School of Agricultural Earth and Environmental Sciences, University of KwaZulu-Natal, Scottsville, Pietermaritzburg, 3209 South Africa; 2 Centre for Transformative Agricultural and Food Systems, School of Agricultural, Earth and Environmental Sciences, University of KwaZulu-Natal, Scottsville, Pietermaritzburg, 3209 South Africa

**Keywords:** Random forest, Remote sensing, Soil organic carbon, Woody encroachment

## Abstract

Woody plants encroachment into grasslands has considerable hydrological and biogeochemical consequences to grassland soils that include altering the Soil Organic Carbon (SOC) pool. Consequently, continuous SOC stock assessment and evaluation at deeper soil depths of woody encroached grasslands is essential for informed management and monitoring of the phenomenon. Due to high litter biomass and deep root structures, woody encroached landscapes have been suggested to alter the accumulation of SOC at deeper soil layers; however, the extent at which woody plants sequester SOC within localized protected grasslands is still poorly understood. Remote sensing methods and techniques have recently been popular in SOC analysis due to better spatial and spectral data properties as well as the availability of affordable and eco-friendly data. In this regard, this study sought to quantify the accumulation of SOC at various depths (30 cm, 60 cm, and 100 cm) in a woody-encroached grassland by integrating Sentinel-1 (S1), Sentinel-2 (S2), PlanetScope (PS) satellite imagery, and topographic variables. SOC was quantified from 360 field-collected soil samples using the loss-On-Ignition (LOI) method and spatial distribution of SOC across the Bisley Nature Reserve modelled by employing the Random Forest (RF) algorithm. The study’s results demonstrate that the integration of topographic variables, Synthetic Aperture Radar (SAR), and PlanetScope data effectively modelled SOC stocks at all investigated soil depths, with high *R*^2^ values of 0.79 and RMSE of 0.254 t/ha. Interestingly, SOC stocks were higher at 30 cm compared to 60 cm and 100 cm depths. The horizontal reception (VH), Slope, Topographic Weightiness Index (TWI), Band 11 and vertical reception (VV) were optimal predictors of SOC in woody encroached landscapes. These results highlight the significance of integrating RF model with spectral data and topographic variables for accurate SOC modelling in woody encroached ecosystems. The findings of this study are pivotal for developing a cost-effective and labour-efficient assessment and monitoring system for the appropriate management of SOC in woody encroached habitats.

## Introduction

The global transformation of mesic grasslands into woody-dominated ecosystems has significantly escalated over the past century (Ratajczak et al., 2012; Van Auken, 2009a, 2009b). The commonly documented drivers of woody proliferation are fire suppression, overgrazing, nutrients availability, climate change and global carbon dioxide enrichment (Kgosikoma & Mogotsi, 2013; Ratajczak et al., 2012). Encroachment of woody plant into grasslands has considerable hydrological and biogeochemical consequences to grassland soils (Honda & Durigan, 2016; Stevens et al., 2017). It reduces the quantity of productive grazing landscapes for wild herbivores and livestock (Aweto, 2024; Ding & Eldridge, 2024; Pinheiro et al., 2022) and reduces the frequency and intensity of grazing and fire, which are key factors maintaining grassland diversity (Ratajczak et al., 2012; Smit & Prins, 2015). According to McKinley et al. (2008), woody proliferation has pronounced impact on below ground nitrogen and carbon pools in grasslands, while Belay and Kebede (2010), Liu et al. (2011) and Mureva et al. (2018) note that the invasion of woody plants into grasslands has a pronounced impact on Soil Organic Carbon (SOC), particularly at an ecosystem level. Alberti et al. (2011), Chiti et al. (2017) and Zhou et al. (2017) note that woody proliferation substantially alters the spatial variability of surficial SOC stocks. However, to date, there is a dearth in literature that has adopted remotely sensed datasets and approaches to establish the accumulation of SOC at deeper soil depths, particularly within woody encroached grasslands.

A study by Zhou et al. (2017) quantified SOC at 120-cm depth and established a high concentration in woody invaded areas compared to pristine grasses. Their study also observed an increase in SOC at deeper soil layers, particularly in woody dominated landscapes. According to Rumpel and Kögel-Knabner (2011), SOC concentration is expected to be higher in deeper soils of woody vegetated landscapes due to higher root litter and decomposition. However, most studies that have investigated SOC in deeper soils of woody encroached grasslands have typically utilized traditional methods that rely on field-based observation that are expensive, labour demanding and protract (Blaser et al., 2014; Mureva et al., 2018). Over the last few decades, advancements in satellite technology and data storage have tremendously revolutionized SOC stocks modelling. Remotely sensed data are pivotal tool for SOC modelling as they offer cost-effective, labour-efficient and time-saving data (Odebiri et al., 2021). Hence, the availability of remotely sensed data promise to revolutionize SOC quantification in deeper soil depths of woody invaded grasslands (Liu et al., 2011; Odebiri et al., 2024; Zhou et al., 2017).

Previous studies investigating the influence of woody invasion on SOC distribution have primarily used multispectral optical sensors including Landsat Mission and MODIS (Venter et al., 2021). However, these sensors are limited by their vulnerability to atmospheric interference leading to decreased accuracy when quantifying SOC in deeper soil layers. Consequently, Synthetic Aperture Radar (SAR) technology has gained traction for monitoring SOC stocks due to its ability to provide weather independent and vegetation sensitive images that are crucial for quantifying SOC in woody invaded landscapes. The European Space Agency (ESA) provides many freely available remotely sensed data with improved spatial resolution. The noticeable advantages of SAR data include the improved spatial and spectral resolutions that can best capture soil-vegetation relationship, making them a feasible and attractive option for quantifying SOC stocks (Zhou et al., 2020). These unique features are important for robust quantification of SOC stocks at deeper soil depths. Additionally, SAR images are not restricted to the visible and infrared portions of the electromagnetic spectrum but possess radar sensors that can be utilized to detect SOC distribution within vegetated landscapes. Also, S2 provides images with thirteen bands that cover the visible near infra-red to short wave infra-red spectral range that is paramount for SOC estimation. Studies by Yang and Guo (2019) and Morais et al. (2023) have reported on the capability of SAR data in modelling SOC stocks in grasslands at different soil layers.

Regardless of these advantages, the capability of SAR data for modelling SOC within woody encroached grasslands has not been fully explored. This is because areas with small geographical extents require images with exceptionally high spatial resolutions, such as PlanetScope (PS) multispectral sensor characterized by 3 m spatial resolution (Koparan et al., 2022). Previous literature shows that a combination of SAR, S2 and PS data is useful in improving the detection of above ground biomass, which eventually informs SOC distribution in deeper soils. According to Wang et al. (2024), local mapping of SOC stocks requires images which finer spatial resolution, such as PS. However, the sensor is only limited to eight bands, and commonly affected by atmospheric interference, hence image scenes might not always be available. Therefore, combining SAR and S2 spectral information with PS data can potentially produce better results when estimating SOC within localized landscapes affected by proliferation of woody plants.

Recently, Machine Learning (ML) algorithms have gained traction for quantifying relationships between SOC and remotely sensed data (Odebiri et al., 2021; Yang et al., 2021; Zhou et al., 2020). Previous literature has proven the value of Random Forest (RF) regression model to quantify SOC stocks within localized heterogeneous ecosystems (Grimm et al., 2008; Pouladi et al., 2019), including woody encroached landscapes (Venter et al., 2021). RF estimates a response variable based on a series of explanatory variable through building a set of regression trees and averaging the outcome. The outcome of all individual trees is averaged to yield a single estimation. RF is easy to implement, and can handle a significant amount of training dataset (Odebiri et al., 2020). Furthermore, RF has a capability to read non-linear correlations through utilizing both continuous and categorical predictor variables, which is fundamental for precise quantification of SOC in woody encroached landscapes. To advance the robustness of the RF, it is fundamental to include topographic factors as one of the predictors of SOC. Environmental variables, such as topographic wetness index, elevation, aspect, and slope, can be combined with remotely sensed datasets to best evaluate the accumulation of SOC stocks in sub-surface soils (Zhou et al., 2020).

Most studies investigating the implication of woody invasion to SOC accumulation have been conducted on surface soils at a depth of 0–30 cm, particularly in grasslands (Liu et al., 2011; Throop & Archer, 2008). Fewer studies have sought to evaluate the accumulation of SOC at deeper soil depth of woody encroached grasslands using remotely sensed data. According to Odebiri et al. (2024), deeper soils (> 30 cm) sequestrate more than half of the total SOC pool. Hence, it is imperative that the extent at which woody encroachment affect SOC is assessed. The lack of comprehensive and conclusive information on the influence of woody proliferation on SOC stocks present an opportunity for further assessment of the phenomenon, particularly in localized grasslands. Consequently, the objective of this study was to estimate soil organic carbon (SOC) at various depths (30 cm, 60 cm, and 100 cm) in a woody-encroached grassland by integrating Sentinel-1 (S1), Sentinel-2 (S2), PlanetScope (PS) satellite imagery, and topographic variables. The study coupled RF, remotely sensed data and topographic variables to model SOC distribution at different soil depths of Bisley Nature Reserve affected by a proliferation of woody vegetation on a grassland. The study also evaluated the spatial distribution of SOC from a wood encroached to a pristine grassland.

## Materials and methods

### Study site description

**Fig. 1:**
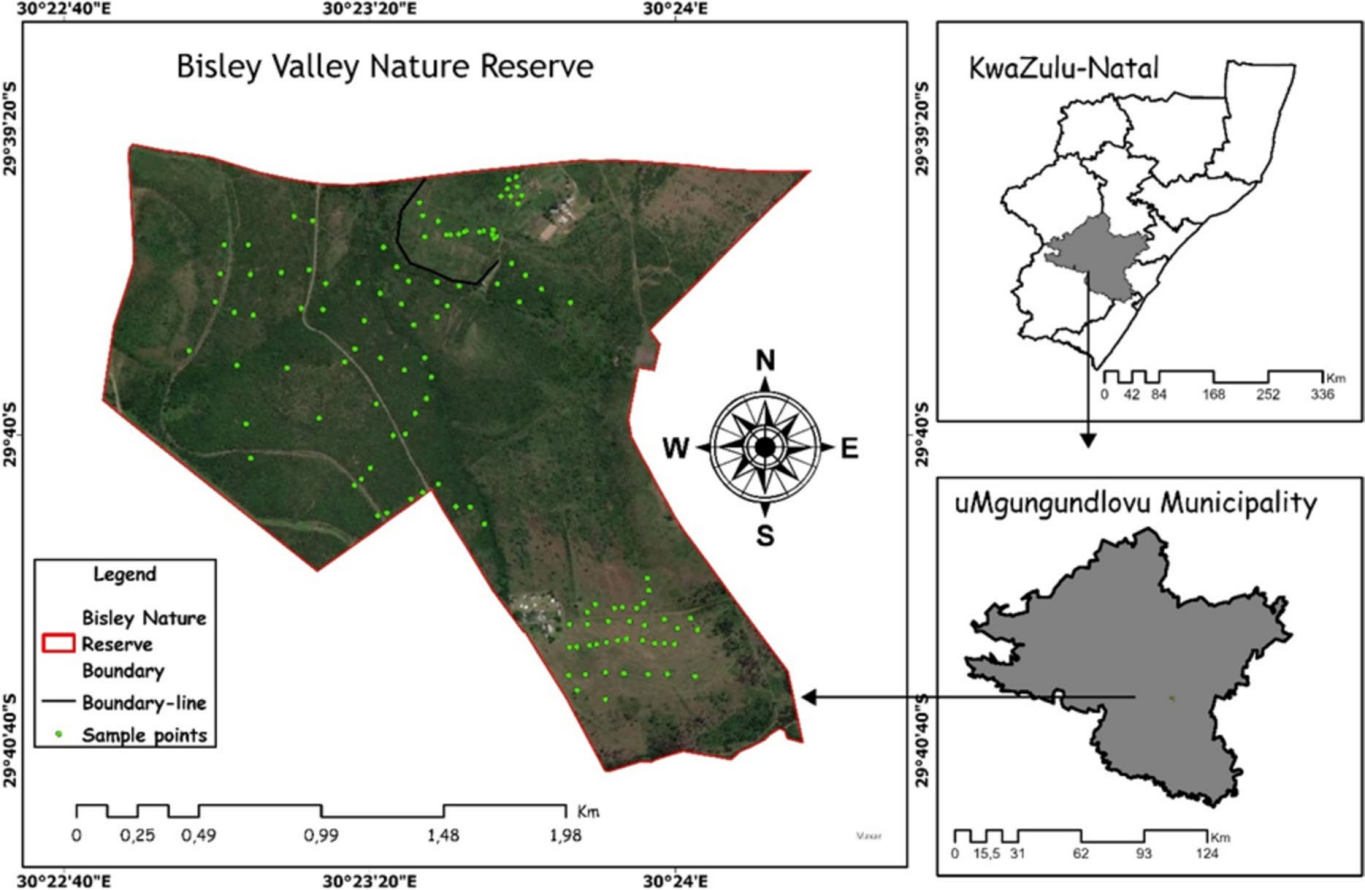
Location of Bisley Nature Reserve in KwaZulu Natal Province of South Africa

The study area is located at Bisley Valley Nature Reserve (29° 39′ 53″ S; 30° 23′ 32″ E), Pietermaritzburg, South Africa (Fig. 1), covering an area of 3.5 km^2^. The nature reserve is identified as transition zone between the Ngongoni Veld and Hinterland Thornveld in KwaZulu-Natal and thus vulnerable to proliferation of woody plants (Kraai et al., 2023). The mean annual precipitation, summer and winter temperature experienced by the reserve are 694 mm, 26 °C, and 17 °C, respectively, and dominated by dry-winter subtropical climate. Observably, *Searsia dentata*, *V. nilotica*, *V. karroo*, and *V sieberiana* are dominant tree species in the area (Kraai et al., 2023), with *E. curcula* and *P. maximum* as common grasses. The wildlife includes giraffe, Impala, Zebra, wildebeest, and a variety of bird species. The soil types that dominate the reserve include Mispah, Dundee, Glenrosa, Clovely, and Avalon, along dolerite as the common rock type. The reserve has an uneven topography with altitude ranging from 700 to 830 m above the sea level. The area is highly encroached by woody trees, thus minimizing the dominance of pure grasses. Although woody trees dominate most of the landscapes, there are few areas dominated by pristine grass patches within the reserve.

### Field data collection

Field sampling was conducted at Bisley Nature Reserve between the 18th and 20th of March 2024. The study employed a purposive sampling strategy to establish three transects that were 622-m long measured from a pristine grassland to a woody encroached grassland. This was done to clearly visualize the transition of vegetation biomass from pristine grassland to woody encroached grassland. At each transect, a total of 40 plots (15-m^2^ each) were established. Within each plot, 1 × 1 m^2^ quadrant was placed every 15 m, and three soil samples collected per quadrant at different soil depths. As a result, a total of 40 soil samples were collected for each depth (30 cm, 60 cm and 100 cm) using a handheld MAC AFRIC 68 CC EARTH Auger Drill. Additionally, utilizing the Trimble Handheld Global Positioning System with a sub meter accuracy, Global Position System (GPS) locations for each plot were recorded. A total of 360 soil samples were collected and subsequently dispatched to the laboratory for analysis.

### Laboratory analysis

The samples were air dried for three days on trays to remove excess moisture. Samples were then sieved through a 2-mm sieve and oven dried for 24 h at 105 °C before analysis. For SOC determination, 1 g of soil was added to glass beakers, placed in a muffle furnace at 360 °C for 2 h, cooled in desiccators at room temperature, and weighted. SOC was calculated as the variance between soil mass before ignition and after ignition, divided by the soil mass before ignition as prescribed by Schulte and Hopkins (1996). All calculated values were tabled in Excel for statistical analysis.

### Image acquisition and preprocessing

The study utilized a combination of radar (SAR), S2 and PS satellite images. The spatial and spectral characteristics of each sensor are presented in Table 1. PS MSI level-3B imagery used in this research was captured on the 8th of March 2024 and downloaded through Planet website (https://www.planet.com/markets/education-and-research/) on the 4th of March 2024 with 5% cloud cover. No image preprocessing was employed for PS imagery as it is supplied atmospherically and radiometrically corrected. PS is characterized by high spatial resolution (3 m) and red-edge band that is critical for SOC monitoring, especially in grasslands with small geographical extent (Matiza et al., 2024).

**Table 1:** Spectral and spatial information of PS, S2 and SAR data

	**PlanetScope**	**Sentinel 1**		**Sentinel 2**
Resolution	3 m	10 m	10 m, 30 m & 60 m	
Band type	Coastal Blue (431–452 nm) Blue (465–515 nm) Green I (513–549 nm) Green II (547–583 nm) Yellow (600–620 nm) Red (650–680 nm) Red edge (697–713 nm) Near-Infrared (845–885 nm)	C-band (VH)	B1 (443 nm), B2 (490 nm), B3 (560 nm), B4 (665 nm), B5 (705 nm), B6 (740 nm), B7 (783 nm), B8 (842 nm), B8a (865 nm), B9 (940 nm), B10 (1375 nm), B11 (1610 nm), B12 (2190 nm)	

SAR data used for quantifying SOC captured on the 05th of March 2024 was downloaded from ESA (https://dataspace.copernicus.eu/) on the 15th of April 2024. SAR products include both Sentinel-1A and Sentinel-1B. According to Geudtner et al. (2014), Sentinel-1A operate in four unique imaging modes which provide C-band with different resolutions. SAR technology is a type of radar that is utilized to build two- or three-dimensional reconstructions of features, including landscapes. This is an active sensor that sends pulses of energy and reads information from the energy reflected by the target object. The sensor provides continuous images that are not affected by clouds, smoke, and haze. SAR offers dual polarisation capability with vertical transmission and either horizontal reception (VH) or vertical reception (VV), and rapid product delivery (Torres et al., 2017). These attributes are critical for continuous monitoring of earth phenomena, including SOC stocks. The Ground Range Detected (GRD) format S1 image of interferometric wide swath mode was pre-processed with ESA’s Sentinel-1 Toolbox in the software SNAP (version 6.4.5). To focus on the study location’s pristine and woody encroached grasses, the data was split into sub-swaths. The image size was also reduced to better processing efficiency. S1 processing routine also incorporated thermal noise removal, Range-Doppler Terrain Correction and geocoding.

S2 image (Level 2B) captured on 15th of April 2024 was downloaded from the Copernicus open access hub (https://dataspace.copernicus.eu/) on the 18th of April 2024. The sensor’s image is characterized by 13 spectral bands with unique spatial resolutions (Table 1). S2 bands range from visible light and near infrared to short-wave infrared. Due to these spectral characteristics, S2 data has an ability to effectively quantify SOC within heterogenous landscapes (Castaldi et al., 2019). S2 image was atmospherically corrected using Sen2Cor processor (V.2.5.5) plugin in SNAP (version 6.4.5) software. The image was resampled to 10 m to maintain the key attributes of S2 data to maximum extent.

### Remotely sensed spectral vegetation indices

Through different band combinations from S1, S2, and PS images, relevant spectral vegetation indices were generated. Previous studies suggest that spectral vegetation indices are paramount predictors of SOC distribution because SOC cannot be directly learned through modelling techniques (Odebiri et al., 2022; Shafizadeh-Moghadam et al., 2022; Zhou et al., 2020). Wang et al. (2021) notes that there is a pronounced relationship between vegetation indices and SOC stocks. As a result, five popularly utilized spectral vegetation indices that have been proven to best predict SOC distribution, were generated from PS, S1 and S2, together with spectral bands and used to predict SOC distribution (Table 2).

**Table 2:** PS, S1 & S2 derived spectral vegetation indices

Index	Formula	Reference
Normalized Difference Vegetation Index (NDVI)	(B8-B4)/(B8 + B4)	(Rouse et al., 1974)
Green Normalized Difference Vegetation Index (GNDVI)	(B9-B3)/(B9 + B3)	(Ahamed et al., 2011)
Red-Edge 1	(Red-Edge/Red)	(Cloutis et al., 1996)
Radar Vegetation Index (RVI)	(8*VH)/ (HH + VV + 2*VH)	(Kim et al., 2011)
Modified Soil Adjusted Vegetation Index (MSAVI)	29 + 1–1*($\sqrt{{\left(29*B1\right)}^{2}-8\left(B9-B5\right)}$)/2	(Wu et al., 2007)

### Topographic variables

Literature shows that there is an association between topographic factors and SOC distribution (Tu et al., 2018; Yu et al., 2020; Zhou et al., 2020). These consist of Elevation, Aspect, Slope, and Topographic Wetness Index (TWI). Topographic residuals were extracted from a SRTM Digital Elevation Model (DEM) image with 30-m spatial resolution acquired from Earth Explore online platform. Using ArcGIS pro (version 3.0), topographic covariates residuals were extracted, and later combined with spectral bands and vegetation indices to quantity SOC distribution in different soil layers.

### Modelling approach

Each observation in our statistical analysis included spectral band values, spectral vegetation indices, and SOC measurements at the plot level that were taken from the relevant plot locations in PS, SAR and S2 images, and topographic variables. The study employed regression techniques to establish the relationship between predictor variables and the field measured SOC. Additionally, using Microsoft Excel and IBM SPPS (version 29), a *t*-test was executed to determine the level of significance between SOC located at the depth of 0–30 cm, 30–60 cm, and 60–100 cm.

### Random forest regression model

The RF algorithm, a non-parametric tree-based machine learning approach, has gained popularity in monitoring and predicting various environmental parameters (Mngadi et al., 2021; Odebiri et al., 2020). As noted by Nabiollahi et al. (2019) and Sreenivas et al. (2014), a distinctive feature of RF is its ability to handle both small and large datasets, making it easy to comprehend and implement. During the training phase, the algorithm first generates a variety of bootstrap samples from the original dataset before randomly selecting various predictors. Furthermore, the algorithm chooses the optimal split among the input variables after each bootstrap has created a regression tree (Singh et al., 2017). Notably, it constructs numerous uncorrelated trees for training dataset, utilizing a random subset comprising two-thirds of all samples, while leaving one-third for validation. Crucially, the optimization of node-size, *mtry*, and *ntree* is essential for achieving optimal predictive performance. The Root Mean Square Error (RMSE) of the training datasets was utilized to optimize the *mtry* and *ntree* to uncover the optimal RF model for quantifying the distribution of SOC (Table 3).

**Table 3:** Random Forest (RF) defined hyper-parameters

Algorithm	Hyper-parameters	Parameter as used	Parameter Description
RF	*Mtry*	25	Number of input variables
	*Ntree*	41	Number of trees
	Node size	1	Number of observations
	Random State	42	Possible combinations of the dataset

### Predictor variable selection

Literature suggests that as the number of input variables increase, RF becomes more complex (Odebiri et al., 2020). For RF model, it is advisable to use a smaller number of predictors to minimize complexity and chances of multicollinearity. Therefore, to prevent multicollinearity, a variable selection method highlighted by Behnamian et al. (2017) was adopted. In this study, the Out-Of-Bag (OOB) error rate with the built in RF model was adopted to eliminate all the input variables that had no contribution towards the accuracy of the model. The backward elimination method was adopted until the best number of predictors was obtained. Consequently, the optimal predictor variables were utilized to generate the final digital SOC distribution map at all selected depths.

### Accuracy assessment

Using a 70–30 holdout validation technique, the RF model’s efficacy in measuring SOC in a woody encroached landscape was evaluated. The total input dataset (N = 120) was separated into 70% (*n* = 84) for training and 30% (*n* = 36) for testing the datasets. To reduce the probability of sampling bias, a tenfold cross validation was adopted. The Root Mean Square Error (RMSE) and *R*^2^ were presented to evaluate the model’s overall performance. RMSE report the difference that exist between the observed and estimated SOC values. The *R*^2^, always between 0 and 100%, indicate the percentage of the response variable difference that is described by the model. Additionally, the significance of each predictor variable to the overall performance of the RF model was assessed using Shapely Additive Explanations (SHAP).

## Results

### Summary statistics

**Fig. 2:**
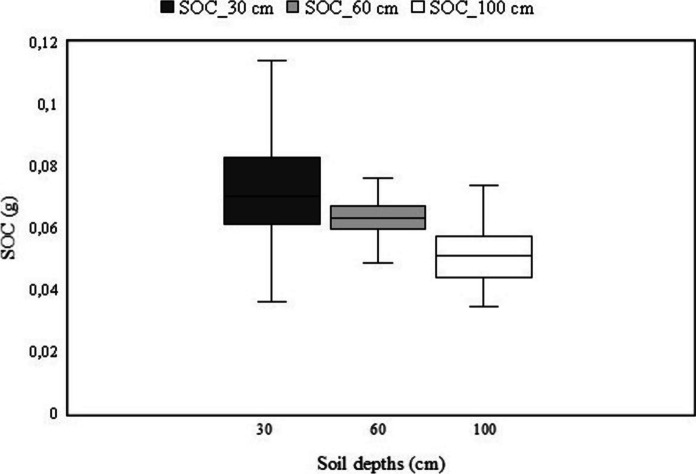
Soil organic carbon distribution at three different soil depths (30 cm, 60 cm & 100 cm)

The summary statistics describing the predicted SOC data at different soil depths are reported in Table 4. SOC data for the three soil depths indicated a normal distribution. The SOC stock varied between 0.037 and 0.095 g in the three soil layers. The Coefficient of Variation (CV) was noted to be low for all the soils depths (< 17%). Results show that the distribution of sample values were negatively skewed for both 30 cm and 100 cm, and positively skewed for 60 cm (Table 4). The results further show that SOC distribution decreased with increasing soil depths and is mostly concentrated at the 30-cm depth (Fig. 2).

**Table 4:** Basic statistics of SOC at different soil depths

Depth (cm)	Minimum (g)	Mean (g)	Maximum (g)	Standard deviation (g)	Standard Error	Skewness	CV (%)	Kurtosis
0–30	0.057	0.077	0.095	0.009	0.002	0.309	11,69	− 0.158
30–60	0.046	0.061	0.069	0.006	0.001	− 1.231	9,84	1.269
60–100	0.037	0.049	0.061	0.008	0.002	0.302	16,33	− 1.330

### Horizontal distribution of SOC

**Fig. 3:**
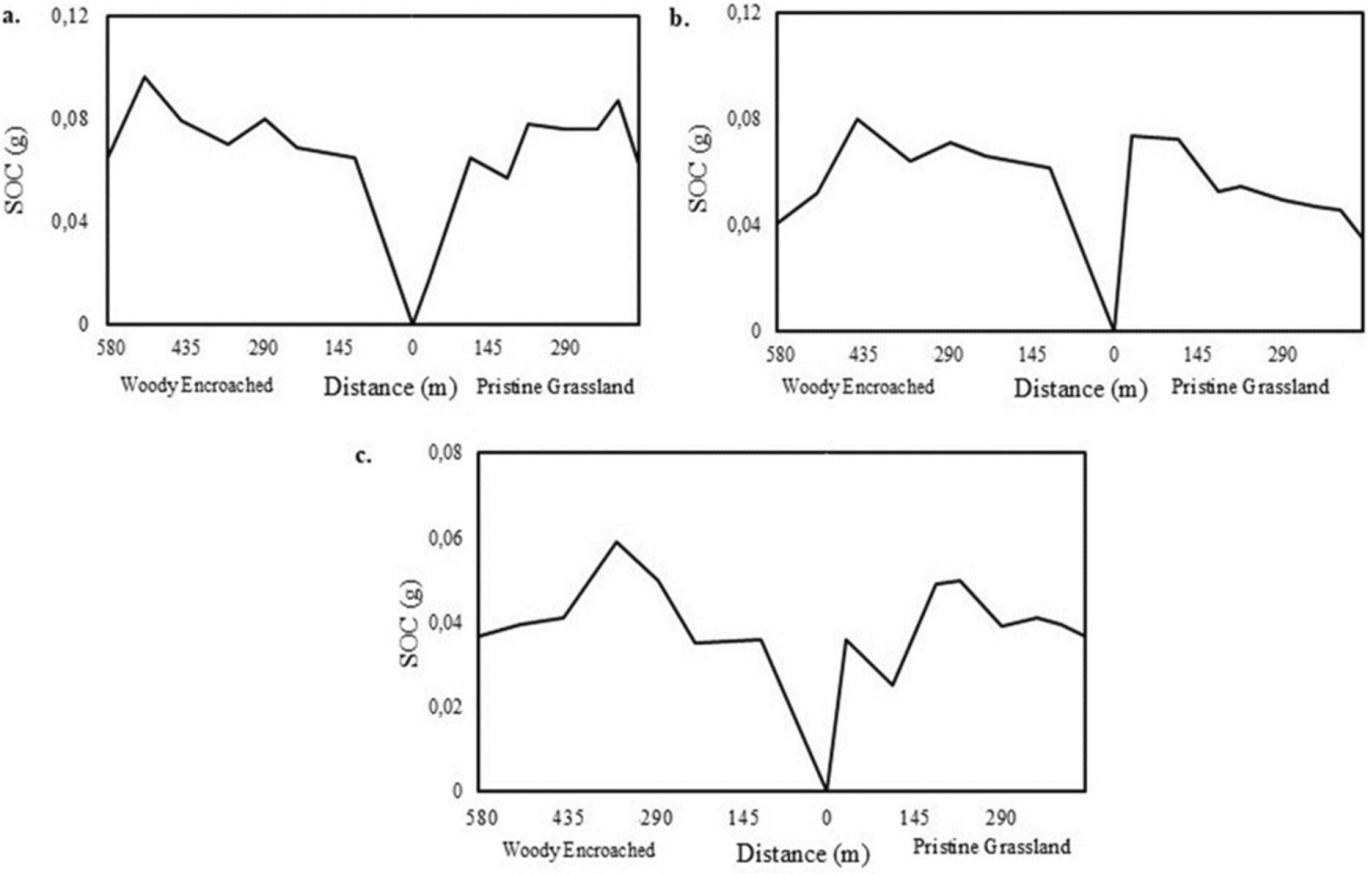
The horizontal distribution of SOC from the boundary towards centre of woody encroached or pristine landscape. **a** SOC distribution at the depth of 30 cm, **b **60 cm, and (**c**) 100 cm

Figure 3 shows the distribution of SOC stock at horizontal distance from the boundary, a centre between pristine and woody encroached landscape, towards either woody encroached or pristine grassland for all the investigated soil depths. Figure 3a, suggest that the amount of SOC increases from the boundary towards the centre of a woody encroached landscape and decreases from the centre toward the edges of the encroached landscape. A similar trend is observed when moving from the boundary towards the centre of a pristine landscape; however, SOC continue to increase towards the edges (Fig. 3a). For Fig. 3b, SOC distribution decreases from the boundary to the centre of the pristine grassland and further decrease toward the edges. These results demonstrate the spatial variability of SOC stocks between a woody encroached and pristine grassland.

### Evaluation and performance of model

**Fig. 4:**
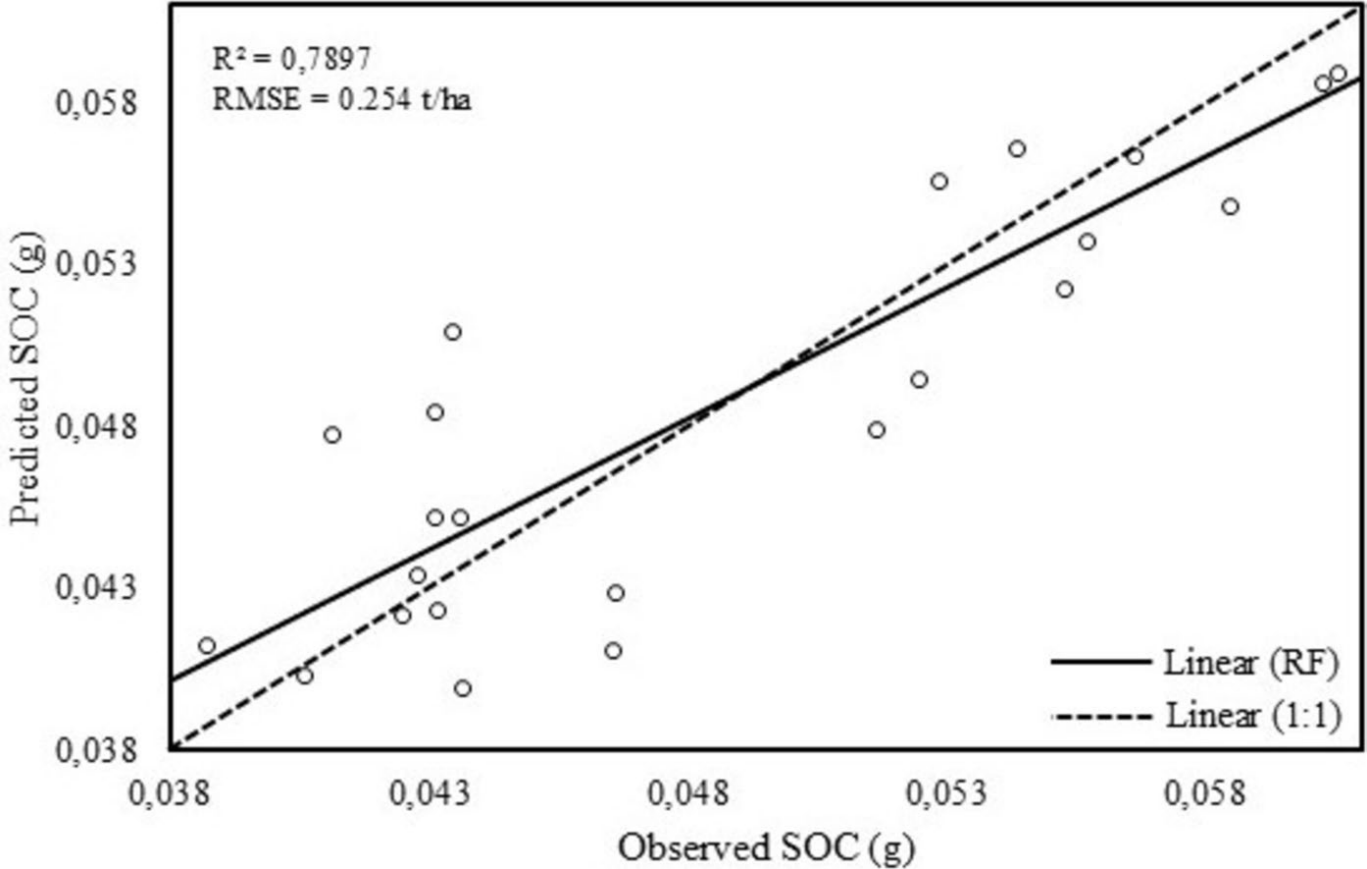
Scatterplot of measured verses predicted SOC values at 0–100 cm depth

The Random Forest (RF) model, utilizing spectral vegetation indices and satellite bands, demonstrated strong performance in quantifying SOC in deeper soil depths of woody-encroached Bisley Nature Reserve exhibiting *R*^2^ of 0.79 and RMSE of 0.254 t/ha. Figure 4 presents the correlation between the estimated and measured SOC values as modelled by the RF algorithm.

### Variable importance ranking

**Fig. 5:**
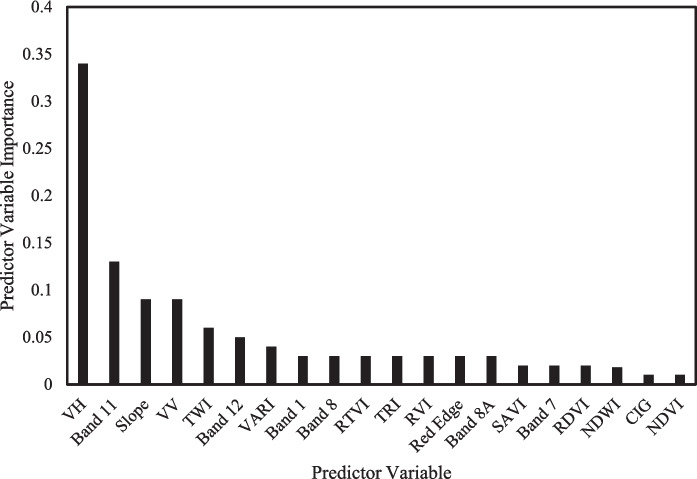
Variable importance ranking for SOC across a woody encroached grassland at 0–100 cm

Figure 5 illustrates the importance of each input variable towards the performance of RF model in quantifying SOC distribution at the depth of 0–100 cm. The Figure reveals that VH, Band 11, and Slope were the most important variables for predicting SOC in deep soils of a woody encroached grassland. A closer look at Fig. 5 shows that VH had the highest contribution while NDVI had the smallest importance and contribution toward the performance of the RF model.

### Spatial estimation of SOC

**Fig. 6:**
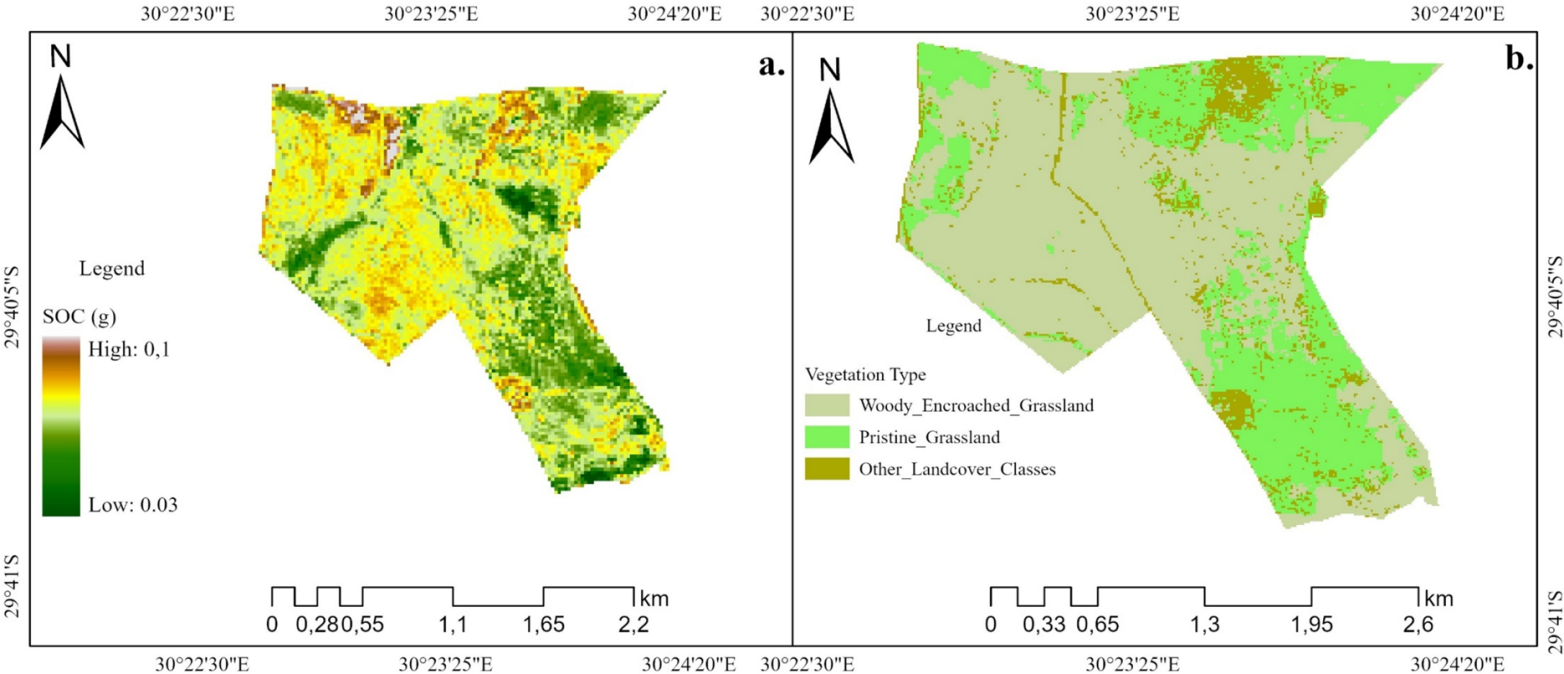
**a** The spatial distribution of SOC for RF model at 0–100 depth. **b** Landcover classification map of the study area

Figure 6a shows SOC accumulation across a woody encroached grassland, while Fig. 6b illustrate different landcover classes located at the study site. A closer look at the maps shows that SOC is predominantly located in central and western parts of the grassland for all the soil depths highly invaded by woody vegetation. Furthermore, landscapes located at the southern and northern parts of the grasslands are dominated by low SOC stocks concentration, and these areas are mostly dominated by grasses. The study clearly shows that SOC is concentrated in areas dominated by woody vegetation, compared to areas under the dominance of grasses for all the investigated soil depths. The maps depict that there is minimal variation of SOC distribution for all the investigated soil depths.

## Discussion

This study used RF and remotely sensed data to model SOC distribution at different soil depths of Bisley Nature Reserve affected by woody proliferation. Our statistical analysis revealed that there is noteworthy variation in SOC distribution between the investigated soil depths. Figure 2 shows a strong resemblance of SOC stock noted at the depth of 30 cm and low accumulation observed at the depth of 100 cm. This finding is consistent with Zhou et al. (2017) who observed high SOC concentration in the 0–15-cm depth increment, and minimal alteration of SOC in deep soils after woody encroachment. According to Zhou et al. (2018), plant roots and root exudates have pronounced impact on SOC distribution because most organic matter in the soil is derived from the root turnover, while Perry (1989) notes that roots of large trees are well entrenched and tougher than immature trees, preventing root turnover in deeper soils. Notably, the bedrock in study area is near the surface, thus restricting root distribution in deeper soils, reducing SOC sequestration (Angst et al., 2018; Kirfel et al., 2019). Furthermore, in terms of SOC stabilization, the results presented by the current study are a response to woody plants that are relatively young (< 100 years)(Kraai et al., 2023). According to Hibbard et al. (2003), it takes more than 200 years for SOC to reach stabilization in deeper soils after woody encroachment. Correspondingly, Zhou et al. (2017) note that a strong SOC resemblance is expected in deeper soils after at least 400 years of woody invasion. For this reason, most woody trees in the study area have not undergone root decay, thus reducing root turnover at deeper soil depths. Hence, SOC accumulation is more amplified in topsoil compared to deeper soils (Yang et al., 2023). Moreover, deeper roots are associated with endurance and gradual turnover compared to roots closer to the surface.

The RF regression analysis revealed that there is a strong resemblance of SOC distribution in woody encroached landscapes compared to landscapes dominated by pristine grasses (Fig. 6). This is because woody encroached areas are dominated by significant litter decomposition, which inform SOC accumulation (McGrath & Zhang, 2003; Odebiri et al., 2022; Zhou et al., 2017). The study also reveals that SOC stocks intensely dominated the central parts of woody vegetated landscapes and declined toward the edges dominated by pure grasses (Fig. 6 and Fig. 3). Similarly, the study by Zhou et al. (2018) established that woody vegetation near the centre is usually older than those closer to the edges, thus explaining the high amount of SOC in the centre of a woody encroached landscape compared to the edges. Previous studies suggest that the establishment of woody vegetation in a grassland greatly amplifies the distribution of SOC pool, suggesting an interconnected relationship between woody encroachment and SOC stocks (Daryanto et al., 2013; Liu et al., 2011). The translocation of SOC and nutrient particles via fluvial, aeolian, and animal transport processes from grass dominated landscapes to areas dominated by woody encroachment, substantially amplifies nutrient and SOC accumulation in woody invaded landscapes (Liu et al., 2011; Okin et al., 2009).

The results also revealed that VH band had the highest importance and contribution in the performance of the RF model (Fig. 5). VH polarization is more sensitive to vegetation density and structural changes and is not affected by weather or clouds (Luo et al., 2024). Even though the frequency of backscattered radar signal of S1 sensor is not a direct measure of vegetation, the backscatter detected in VH band is directly linked to the above ground vegetation (Duarte et al., 2024). Therefore, VH cross polarization band provide crucial information about the physiological state and changes of above ground biomass which relate to the accumulation of SOC stocks. The study by Santos et al. (2023) also discovered VH polarization to be the most important variable explaining SOC distribution for RF model since it has the ability to penetrate the ground. Additionally, topographic derivatives, slope and TWI, were among the most significant input residuals for detecting the concentration of SOC at deeper soil depth, across a woody encroached grassland. Similarly, Zhou et al. (2020) observed that topographic variables obtained high accuracy compared to S1 and S2 data when estimating SOC pool. The study by Barbosa et al. (2014) suggests that slope is the key determinant of vegetation density and thus explaining SOC pool. According to Tziachris et al. (2019), toe-slope positions are associated with high SOC concentration, due to high vegetation biomass, compared to mid-slopes, and thus explains the robustness of the slope in explaining SOC distribution.

The integration of topographic variables, S1, PlanetScope and SAR data performed well in modelling the horizontal and vertical accumulation of SOC pool across the study area (Table 4). The current study concludes that combining SAR and PlanetScope with topographic factors improves the prediction of SOC within a woody encroached grassland. Moreover, the study indicates that the use of RF regression model, based on combined remotely sensed input variables provide a reliable and effective methodology for modelling SOC in woody encroached ecosystems (*R*^2^ of 0.79 and RMSE of 0.254). The results demonstrated the ability of RF to select the best input variable for producing SOC distribution maps (Fig. 6). One of the shortcomings of RF regression algorithm is failure to deal with complicated and heterogeneous data (Odebiri et al., 2020). However, the current study utilized less complicated dataset with few predictor variables, thus explaining the success of RF model in predicting SOC.

Our results have revealed more concentration of SOC in woody encroached landscapes compared to grass-dominated landscapes (Fig. 6). Previous literature has revealed that SOC stocks promote productivity and stability in grasslands and is crucial for climate change mitigation (Conant, 2010; Ghosh & Mahanta, 2014; Tessema et al., 2020). However, its role on reducing and degrading grasslands has not been well documented, particularly after woody plants encroachment. Van Auken (2009a, 2009b) and Wolkovich et al. (2010) note that due to increased soil fertility and SOC enrichment in woody encroached landscapes, the dominance of woody plants is most likely while grasses degrades. Hence, its adverse impact on grasses overrides its positive impacts. Additionally, the study has established more concentration of SOC stocks in central parts of the reserve, hence priority should be given to these landscapes for effective management of the reserve and reduction of woody invasion.

## Conclusion

We used topographic factors, PlanetScope and SAR data to quantify the accumulation of SOC in Bisley Nature Reserve using ML techniques. The study established that integration of spectral information with vegetation indices and physical variables provide valuable information for monitoring SOC distribution in woody encroached grasslands. RF model performed well in estimating SOC in the study area, e.g. exhibiting *R*^2^ of 79.0 and RMSE of 0.254 t/ha. We conclude that freely and accessible SAR and PlanetScope data and topographic factors provide more opportunities to quantify SOC stocks in grasslands. The study is beneficial to reserve managers and policymakers to make informed decisions on conserving the nature reserve. The methodology presented by the study is a cost-effective and time-efficient procedure of monitoring SOC distribution across woody encroached grasslands. Reserve managers can use the insights of the study to establish effective land management patterns to preserve and maintain SOC pool. We, however, suggest that future research explore the combination of bioclimatic variables with remotely sensed data to model SOC stocks in woody encroached landscapes.

## Data Availability

No datasets were generated or analysed during the current study.

## Declarations

### Suggested reviewers

1. Dr Cletah Shoko (University of the Witwatersrand, Johannesburg) cletah.shoko@wits.ac.za.

2. Dr Terrence Darlington Mushore (University of Zimbabwe), tdmushore@gmail.com.

3. Dr James Magidi (Tshwane University of Technology), magidijt@tut.ac.za.
